# Plane Fitting in 3D Reconstruction to Preserve Smooth Homogeneous Surfaces

**DOI:** 10.3390/s22239391

**Published:** 2022-12-01

**Authors:** Yanan Xu, Yohwan So, Sanghyuk Woo

**Affiliations:** 1 Affiliated Research Institute, Metabank Inc., Daejeon 34430, Republic of Korea; 2 Department of Media and Visual Communications, Hannam University, Daejeon 34430, Republic of Korea

**Keywords:** weakly-textured, homogeneous surface, plane fitting, 3D reconstruction

## Abstract

Photogrammetric techniques for weakly-textured surfaces without sufficient information about the R (red), G (green) and B (blue) primary colors of light are challenging. Considering that most urban or indoor object surfaces follow simple geometric shapes, a novel method for reconstructing smooth homogeneous planar surfaces based on MVS (Multi-View Stereo) is proposed. The idea behind it is to extract enough features for the image description, and to refine the dense points generated by the depth values of pixels with plane fitting, to favor the alignment of the surface to the detected planes. The SIFT (Scale Invariant Feature Transform) and AKAZE (Accelerated-KAZE) feature extraction algorithms are combined to ensure robustness and help retrieve connections in small samples. The smoothness of the enclosed watertight Poisson surface can be enhanced by enforcing the 3D points to be projected onto the absolute planes detected by a RANSAC (Random Sample Consensus)-based approach. Experimental evaluations of both cloud-to-mesh comparisons in the per-vertex distances with the ground truth models and visual comparisons with a popular mesh filtering based post-processing method indicate that the proposed method can considerably retain the integrity and smoothness of the reconstruction results. Combined with other primitive fittings, the reconstruction extent of homogeneous surfaces can be further extended, serving as primitive models for 3D building reconstruction, and providing guidance for future works in photogrammetry and 3D surface reconstruction.

## 1. Introduction

Image-based 3D reconstruction is a method of obtaining a 3D model with a series of images captured by a camera around a target object, as well as obtaining 3D coordinates on the basis of the triangulation principle [1]. The avenue of deep learning and the availability of low-cost scanning devices have greatly enhanced the accuracy and popularity of 3D reconstruction technologies, making it increasingly important in commercial and research domains such as object recognition, 3D modeling and animation, extended reality, industrial control, etc. [2]. 3D reconstruction depends primarily on a well-known pipeline that is performed based on the SFM (structure from motion) algorithm with a combination of the MVS framework. Traditional MVS-based photogrammetry pipeline consists of camera motion estimation, feature extraction, image and feature matching, bundle adjustment, and finally 3D point estimation [3], in which, the features to be extracted are keys to the success of the pipeline, which are derived from the R, G, and B information of the image pixels. The more features detected, the more detailed the description of the object, the more accurate the estimated camera motion, and the denser the resulting surface [4].

So far, traditional MVS-based 3D reconstruction algorithms remain a major part of the research and application, and most objects or scenes can be performed accurately, provided that the photos can be captured in high quality [5]. However, MVS-based 3D reconstruction can be an issue for surfaces with homologous, weakly-textured, transparent, reflective regions, and is vulnerable to the background [6]. Various studies have attempted to tackle these deficiencies, such as augmenting image contrast in detail [7], estimating valid depth pixels [8], or optimizing the capture environment [9], but the trade-off between enhanced features and noise control continues to be challenging. Additionally, the output of typical dense 3D reconstruction approaches is often not compact and simple enough for industry applications [10]. For example, in urban reconstruction, vegetation and ornaments are mostly too noisy to directly describe the shape of the building with a scanned 3D model, so extensive artificial post-processing is required.

This paper addresses the problem of the 3D reconstruction of objects with homologous planar surfaces from aspects of feature enhancement and noise reduction. Particularly, with a view to minimizing manual post-processing of 3D models, it investigates the multi-plane fitting technique to confirm that all points are on an absolute plane to facilitate *ROI* adaptation and surface smoothing. The plane fitting-based 3D reconstruction method to preserve smooth homogeneous surfaces is proposed for the purpose of modeling urban buildings or interior structures. The reason behind this is that most urban buildings or interior structures somehow combine various planes, which tend to be low-textured or homogeneous, such as Manhattan-like structures. Another benefit of plane fitting is it can also automatically populate the planar bottom of the model in the absence of images from below. In contrast to many works that favor the most images and the best reconstruction, the proposed approach is able to deal with small samples and is robust to images with noisy backgrounds and poor illumination as well.

The rest of this paper is organized as follows. First, a review of related technologies is provided. Followed by a detailed address of the proposed method for reconstructing a smooth and enclosed homologous planar surface based on MVS. The results of the experimental evaluation in relation to other state-of-the-art methods are subsequently given. the conclusion is set forth in the last section.

## 2. Related Works

Fusing depth maps into a point cloud or a volumetric representation of the object is a common approach for MVS-based 3D reconstruction, and has been widely used due to its efficiency and scalability [11,12]. Popular depth estimation methods such as semi-global matching [13], and PatchMatch-based algorithms [14,15] are relatively mature, but struggle to handle surfaces lacking reliable data for depth estimation, such as smoothness, low-texture, and homogeneous regions. To overcome this barrier, Schops, T., et al. [12] introduced higher-level scene understanding constraints to facilitate the propagation of correct depth estimates between adjacent pixels. Aldeeb, N. H. and Hellwich, O. [7] attempted to amplify local contrast rather than local noise to highlight surface features. These algorithms incline to the artificial processing of collected images, thereby decreasing the automation of 3D reconstruction. The development of deep learning in recent years has brought a new impact to 3D reconstruction [16]; however, most techniques tend to the generation of depth values beyond typical primitive detection, and hence cannot achieve the reconstruction of objects or scenes directly.

In urban or indoor environments, most object surfaces follow simple geometric shapes, such as planes, spheres, and cylinders, which can serve as strong local shapes to treat noise and outliers, as well as other common problems in 3D reconstruction, such as inconsistency and missing data [17]. As the most common approach to detect typical primitives in the input point cloud, primitive fitting is widely used in urban or indoor 3D reconstruction, particularly in building modeling. Most studies tend to obtain approximate polyhedral shapes with the inliers after particular primitive fitting [18,19]. Works that aim to enforce the surfaces to be consistent with prior geometries are given as well [10,20].

To realize a homogeneous surface reconstruction using the most popular primitive information and the generic MVS-based 3D reconstruction pipeline based on depth estimation, the difficulty and novelty involved in this paper lie in the detection of points of interest, and surface smoothing to minimize manual post-processing.

Capturing setup. In addition to the manual processing of image properties such as contrast before feeding to the pipeline [7], the question of how to realize the most automated reconstruction process with the most convenient installations is analysed by numerous surface reconstruction studies [21,22,23,24,25]. Projecting artificial patterns onto the object surface is a common method for homogeneous surface reconstruction, but is susceptible to projector luminosity [23,24], so high-quality scanners and lighting equipment are often required [25]. Furthermore, accurate projection on complex surfaces must be cumbersome. Instead of projecting, the proposed approach places several small, easily removable stickers on homogeneous regions to help with feature detection and simplify capture setup.

In many cases, limited interior space makes it difficult to take photos of objects from multiple angles by moving the camera. At this point, a turntable that can carry an object and rotate is widely adopted [26,27]. The advantage of the turntable is adapting the object to all-around shooting, thus capturing all aspects of the object in a full loop [28]. The most popular purpose of a turntable tends to impose restrictions on the shooting environment from the viewing angle to illumination, which requires specialized customization. The acquisition of the *ROI* either depends on complex axis calibration or requires additional means, such as masking or fiducial markers, which are relatively complex and not always available in common pipelines.

In this paper, the turntable is proposed to help capture model images from all angles in a limited interior space, which can be any rotating plane convenient for indoor use. Another benefit of the turntable is the ability to restore the bottom planar surface with the aid of plane fitting in the absence of bottom photos, which can also reduce the sample size to some extent. Additionally, due to the limited features of describing homogeneous, weakly-textured surfaces, they are more sensitive to background noises such as shadow, reflection, and camera position. Consequently, a plain color background with no distinguishable features is considered ideal.

Feature extraction. SIFT algorithm is the most well-known feature detection method, with a stable invariance in image transformations. However, the Gaussian blur algorithm used by SIFT to build the scale space can lead to the loss of edge information, which is unfavorable for the reconstruction of homogeneous surfaces dependent on edge features [29]. Li, Zinuo, et al. [5] proposed a Matting-SFM algorithm to eliminate the background of the input samples and highlight the edges, but this requires sufficient texture on the object surfaces. Zhang W. [29] used a Canny edge detection algorithm to correct unstable SFM edge points, but this is only valid for detectable descriptors.

In contrast, the AKAZE algorithm considers both the nonlinear and anisotropic scale space, and the local features around the edges are more easily retained than SIFT [30,31]. Based on the prior method [31], a combination of SIFT and AKAZE to extract the most detailed features possible is proposed.

Smooth surface reconstruction. 3D surface smoothing methods range from the simplest Laplacian-based method [32] to geometry-based filtering techniques for triangular mesh [33], but most of them are dedicated to the post-processing of the generated meshes and are unclear on the parameters. Resampling point clouds in an evenly distributed manner may also help to smooth surfaces to some extent [34]. Starting with a similar idea, it is proposed to redistribute the point cloud by plane fitting to generate a smooth planar surface directly without further tedious post-processing.

In works of shape reconstruction from point clouds, the Poisson reconstruction is superior to other methods in constructing smooth surfaces, given the smoothness properties inherited from the basis functions used in the Poisson equation [35]. However, it is sensitive to point normals. Visibility-based graph cut is typically used to label the inside or outside of a given surface [36], but after repositioning, the point visibility information derived from given camera-point correspondences will be lost. The proposed method of computing the position of the planes constituting the surface relative to the center of gravity of the point cloud can easily orient the point normals.

## 3. Materials and Methods

**Figure 1:**
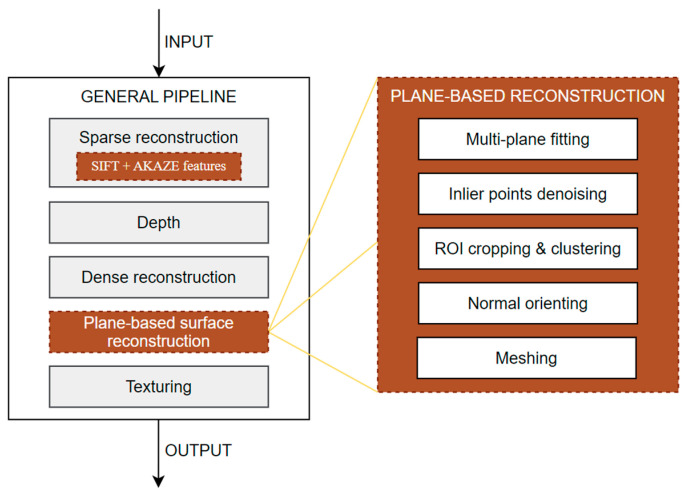
Flowchart of reconstruction process.

The flowchart of the reconstruction process is depicted in Figure 1; the pipeline is introduced based on AliceVision [36], in which image features are first extracted for camera calibration and sparse 3D point inferring. The depth value of each pixel is then retrieved based on a semi-global matching approach [13]. The estimated depth maps are then fused into a global dense point cloud to create a dense geometric representation of the object. The goal of the proposed method is to process the dense point cloud before the generation of meshes, by aligning the points with the fitted planes for a smooth surface, which may be available for industrial application with no further manual post-processing. What needs to be carried out is to drop the visibility information of the original points [37] in the conventional MVS-based reconstruction pipeline, and instead perform a 3D triangle mesh extraction by repositioning the dense point cloud and revising the orientation of the point normals.

### 3.1. Feature Extraction

In sparse reconstruction, a feature extraction method combining SIFT and AKAZE is proposed to ensure that the features are not affected by the image rotation, translation, and scale during acquisition, and to help retrieve connections in small samples. AKAZE can compensate for the edge information smoothed out by Gaussian descriptor in SIFT, and SIFT is robust to a large number of variables, including background, light, and affine rotation, thanks to a well-studied theoretical foundation.

**Figure 2:**
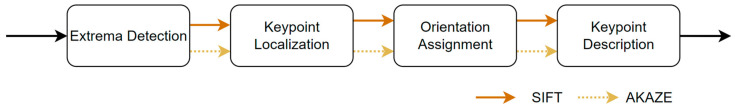
Flowchart of feature extraction.

As shown in Figure 2, the combination of SIFT and AKAZE can be achieved by four steps: extrema detection, keypoint detection, orientation assignment, and keypoint description. Feature detection based on SIFT and AKAZE performs in parallel, with each step depending on different algorithms or methods, specifically referring to [30,31]. For example, when creating the invariance of extrema to image scale and orientation, the Gaussian scale and non-linear scale-space are built by SIFT and AKAZE for image blurring, respectively. The purpose of keypoint localization is to remove unstable extrema from noise, with the remaining as considered initial keypoints. The orientation assignment aims to assign orientations to each keypoint so as to obtain the image rotation invariances. An adapted descriptor is then used to represent the keypoints. The final features are obtained by the merger of normalized keypoints vectors.

**Figure 3:**
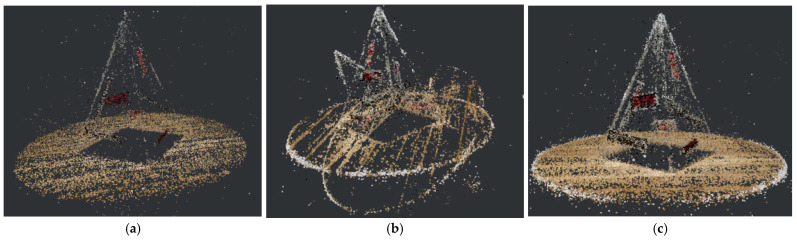
Results of SFM based on different feature extraction algorithms: (**a**) SIFT-based (22,273 points); (**b**) AKAZE-based (31,477 points); (**c**) SIFT + AKAZE (69,028 points).

Figure 3 shows the sparse points generated by SFM. SIFT (a) provides considerable robustness, especially for affine rotation [38], and outperforms AKAZE (b) in describing textured regions. AKAZE (b) preserves greater edge details than SIFT (a), and the proposed method (c) can be seen as an optimal solution by incorporating the benefits of SIFT and AKAZE, and offsetting the shortcomings.

### 3.2. Multi-Plane Detection

**Figure 4:**
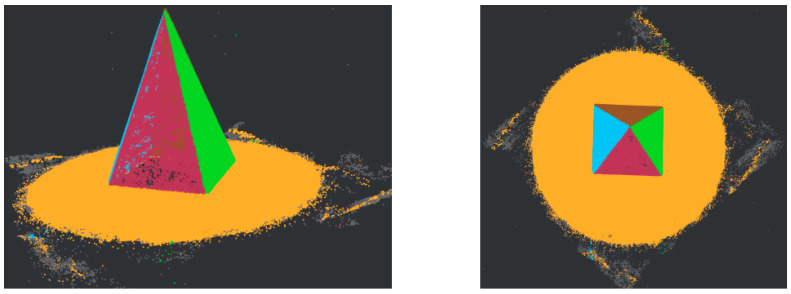
Multi-plane detection.

A RANSAC [39] based approach is used for multi-plane detection. This is performed by repeatedly detecting the plane containing the most points in the point cloud until the remaining points are less than the minimum number of points available for clustering $n_{min}$. The inliers supporting a plane are defined as the points within a maximum distance $d_{max}$ to the plane. In general, $n_{min}$ determines the area of a plane, while $d_{max}$ restricts the tolerance of inliers. In other words, smaller values of $n_{min}$ are susceptible to microplane detection and thus retain more planar details on the surface. The $d_{max}$ concerns the compactness and smoothness of the reconstructed surface. Note that, given the confidence of the RANSAC algorithm is correlated with problem-specific thresholds, if $d_{max}$ is too large or too small, it may result in either a mutual fusion of different planes or an excessive aggregation on the same plane. A maximum number of iterations $iterra_{max}$ is used to terminate the RANSAC loop, to prevent memory overflow when all remaining points are more than $n_{min}$ but cannot constitute a desirable plane. The default values of $n_{min}$, $d_{max}$ and $iterra_{max}$ are defined as 0.01n, 0.01, and 100, respectively, where n represents the point cloud size. As shown in Figure 4, multiple planes are detected in an input point cloud, with points on the same plane marked with the same color, and gray points identified as outliers.

### 3.3. Inliers Denoising

The RANSAC-based plane fitting method is to select the model parameters corresponding to the plane containing the largest number of inliers in a disordered point cloud in each fitting loop, and then remove the distant points from the planes, namely outliers. The inliers are evenly distributed on either side of the plane at a distance not exceeding $d_{max}$, and are not all located on the detected planar surface. Triangulation using these points directly may result in depth differences between the vertices, which will be responsible for generating potholes on the reconstructed surface.

**Figure 5:**
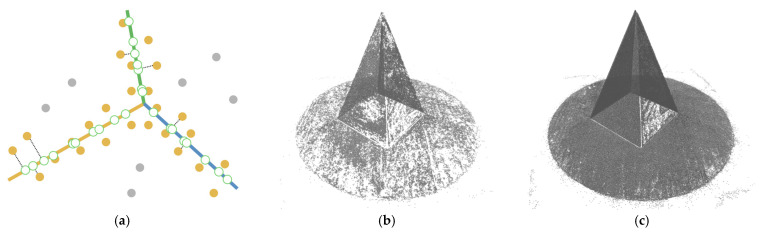
Inlier denoising: (**a**) Plane-based projection; (**b**) point cloud with noise erasure direct (210,088 points); (**c**) point cloud after projection-based denoising (471,109 points).

Regarding the inliers not on a plane model as noises, denoising is necessary for a smoother surface. It is natural to consider erasing these noises and leaving only points on the plane, but at the cost of a reduction in the density of the point cloud. It is obviously not desirable for homogeneous surfaces, given that the failure to reconstruct such surfaces with no significant characteristic changes is largely due to the shortage of points. In contrast, it is proposed to project all inliers onto the corresponding planes; that is, to replace the point with its projection on the plane, so as to maintain the initial density of the point cloud. As illustrated in Figure 5a, the dark yellow dots represent the initial inliers, which should be replaced by the corresponding projection points (green hollow dots) on the plane (solid lines), and the points which cannot be fitted by planes are considered to be outliers (gray dots). Figure 5b shows that by directly erasing the noise from the inliers, the density of the point cloud is reduced by about 50%, while the plane-based projection can maintain a density similar to the original point cloud (475,470 points), removing only outliers, as shown in Figure 5c. Note that, in many cases, plane fitting cannot guarantee that planes pass through any point, so a minor distance deviation of 1 × 10^−3^ is allowed in obtaining (b). In addition, inlier denoising does not take into account the presence of overlapping projection points, because the sharing coordinates do not affect the mesh results. However, as is known, outliers are usually introduced by ambient noise, so the removal of outliers can improve the reconstruction precision to some extent.

### 3.4. *ROI* Cropping and Clustering

The *ROI* refers to areas of a dense point cloud that belong to the homogeneous surface of the target object. For images collected by the turntable, due to the texture significance of the tabletop portion, it is easier to detect and describe in relation to the homogeneous portions in reconstruction, and the points will be quantitatively more prominent than the *ROI* in number. Therefore, given the plane set after plane fitting $P=\left\{p_{1}\right.,\cdots ,\left.p_{n}\right\}$, the largest one $p_{max}$ can be considered as the plane in which the tabletop is located, and should be eliminated. Due to the lack of images from the bottom of the object, the point cloud needs to be augmented to sample the bottom points. It is proposed to project all points onto $p_{max}$ to obtain a plane $p_{all}$ with the same density as the point cloud, and after removing the overlapping points of $p_{all}$ and $p_{max}$, the intersection $p_{roi\_bottom}$ of the *ROI* and tabletop can be retrieved. Therefore, the following plane set $P_{roi\_raw}$ can roughly describe the *ROI*:

(1) $$P_{roi\_raw}=\left\{p_{1},\cdots ,p_{n},p_{roi\_bottom}|p\neq p_{max}\right\}$$

The premise of separating the *ROI* from the inevitable background and edge disturbances is that each point is classified into specific groups with similar properties; that is, clustering. Several popular clustering algorithms are available for data analysis, in which, an unsupervised clustering machine-learning algorithm called DBSCAN (density-based spatial clustering of applications with noise) [40] is proposed for *ROI* segmentation. The biggest strength of DBSCAN over others is the capability to detect clusters of arbitrary shapes, benefitting from the density-based method of cluster detection. While most center-based cluster definition algorithms, such as k-means and mean shift, are underperforming for non-spherical shaped clusters [41]. Since the proposed method is intended to exclude distribution-agnostic ambient noise, the clustering algorithm should have the ability to find the different numbers of groups. Another superior aspect of DBSCAN that supports the proposed method is the good performance in recognizing noise and outliers, and the non-essential assumption regarding the number of clusters, which is unpredictable in the point cloud. After clustering $P_{roi\_raw}$, the cluster $c_{max}$ which contains the most points is considered the *ROI*.

(2) $$ROI=\left\{point_{1}\right.,\cdots ,\left.point_{n}\right\}$$

where, $point\in c_{max}$ and $c_{max}\in P_{roi\_raw}$.

**Figure 6:**
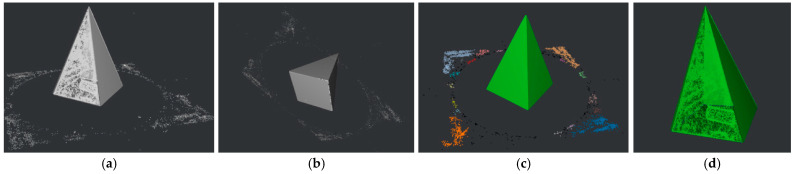
ROI cropping and clustering: (**a**) $p_{max}$ removal; (**b**) $p_{roi\_bottom}$; (**c**) clustering; (**d**) *ROI*.

Figure 6 illustrates the interception process of the *ROI*. (a) Shows the result of $P_{roi\_raw}$, the $p_{roi\_bottom}$ is given in (b) from a bottom perspective, (c) represents the expected clusters from the density distribution, and (d) is the final *ROI*.

On the other hand, if the images are taken off a turntable, the tabletop processing can be ignored. The downside, however, is that clustering denoising may not be available if the noisy points in the interface of the model and its supporting tools are very close to the *ROI*. In this case, alternative post-processing is necessary. This is why a turntable is suggested, which can minimize manual post-processing of the 3D model.

### 3.5. Normal Orienting and Meshing

The screened Poisson-based surface reconstruction method [42] is used to create a smooth 3D geometry from an unstructured point cloud. It is the most popular technique but is sensitive to the normal of the input points [35]. RANSAC-based plane fitting gives the initial orientation of the normal vector, which determines the inside or outside of a plane, and all points on the same plane should share the same orientation as well. To surround an enclosed surface with the detected planes, the visible side has to be external; that is, the normals of all points should point outward from the model. The main idea to determine the target orientation of point normals depends on the relative location of the center of gravity of the point cloud and plane models. Let $p_{center}=\frac{1}{n}\overset{n}{\underset{i=1}{\sum }}ROI_{i}$, as the center of gravity of the point cloud and $N_{plane}=\left\{normal_{plane_{1}}\right.,\;\cdots ,\;\left.normal_{plane_{n}}\right\}$, as the normal of each plane model obtained by RANSAC, define target point normals as $N_{ROI}=\left\{normal_{point_{1}}\right.,\;\cdots ,\;\left.normal_{point_{m}}\right\}$, where n and m refer to the number of planes and points in the *ROI*, respectively. If $n_{model}$ points to $p_{center}$, it points inside the model, then the target normal of points on that plane should take its inverse value. Conversely, if $n_{model}$ points out of $p_{center}$, it points out of the model, then the relative target point normals should be in the same orientation. Thus, it has:

(3) $$\begin{array}{c} normal_{point_{i}}=\lambda normal_{plane_{t}},\\ \lambda =\left\{\begin{array}{cc} 1, & p_{center}\cdot normar_{plane_{t}}<0\\ -1, & p_{center}\cdot normal_{plane_{t}}>0 \end{array}\right. \end{array}$$

where, $point_{i}$ is the *i*-th point located on the *t*-th plane $plane_{t}$ of the *ROI*.

**Figure 7:**
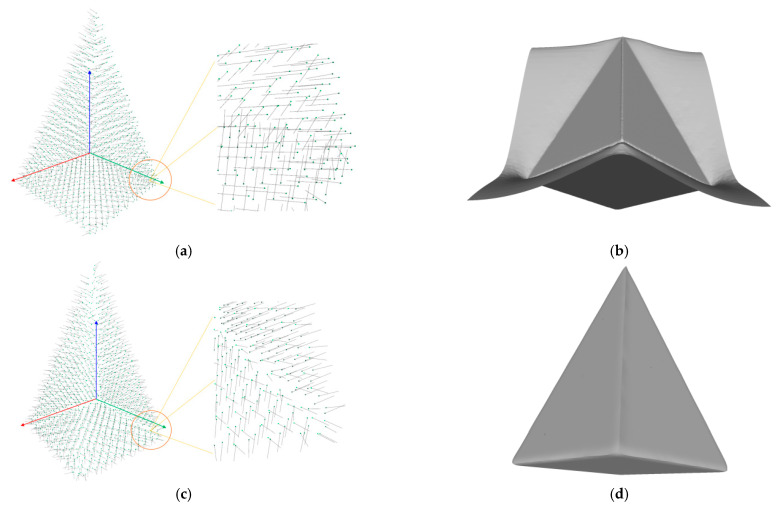
Normal orientation and meshing result: (**a**) default point normals; (**b**) mesh before normal orientating; (**c**) point normals after orientating; (**d**) mesh after normal orientating.

Figure 7a draws the point normals according to each plane mode ax+by+cz+d=0 after plane fitting. As can be seen, certain normals appear within the model, resulting in an unexpected Poisson surface, as shown in Figure 7b. In Figure 7c, after correction, the normals pointing toward the center of gravity will be reversed, and the *ROI* surface can eventually be reconstructed given in Figure 7d.

## 4. Experimental Evaluation

Commonly used photogrammetry applications focus on restoring the model shape without paying much attention to detailed features such as surface smoothness, so they often rely on further post-processing to obtain useful models for industrial applications. The state-of-the-art photogrammetry software, represented by Agisoft Metashape [43] and Meshroom [36], is not only widely used in urban reconstruction, but can also deliver better results in 3D object reconstruction than mobile-based 3D scanners [44].

In this section, four models with homogeneous surfaces are designed to simulate common architectural structures, for which photogrammetry-based 3D reconstruction is performed. By comparing the reconstruction results from different methods, the contribution of the proposed method in improving the automation of 3D reconstruction is underlined. Table 1 lists the specifications of the experimental system.

As stated in Section 2, an object with a completely homogeneous surface cannot be reconstructed in view of the absence of descriptive features for extraction and comparison. The experiment simplifies the most popular pattern projection approach by attaching several easy-to-remove stickers to the model surface. All models were photographed with the 4.25 mm f/1.8 dual-wide camera on an iPhone 11. The reason lies in the simplicity of acquisition and operation of smartphones that are available to individuals, indicating the tolerance of the proposed method for image quality; a professional camera with high specifics will certainly help to enhance the precision of outputs. All images were taken under natural light conditions indoors, and a wooden turntable with an area ratio of about 4:1 to the model bottom was used. The angle of each rotation is not mandatory, as long as the adjacent images satisfy the minimum 60% side overlap and 80% frontal overlap, to fully cover the model from all perspectives.

Table 2 presents the meshing results for each model, with the number of input images varying from 35 to 70, depending on the different shapes of the models. The output accuracy of Metashape is set to medium, and Meshroom is run in default mode. The results reveal that each method can yield a relatively ideal result when the background is fairly simple. Nonetheless, the proposed method can effectively smooth the planar surface while ensuring the integrity of the model shape visually. In addition, if the plane where the tabletop is located can be easily identified after plane fitting, then the *ROI* can be segmented using a density-based clustering method, so as to minimize manual 3D model post-processing and improve reconstruction efficiency. It is worth mentioning that the proposed method is much more robust to affine rotation, in reference to the results of *model-2*.

Table 3 lists the outputs of another dataset, where the input images were collected under the same environmental conditions, but not in a completely blank background; the number of input images varies from 35 to 52. Unlike the rich-textured objects with sufficient characteristics to distinguish between foreground and background, homogeneous surface reconstruction is extremely demanding for high image quality, as both foreground and background features will be detected in MVS-based 3D reconstruction. To this end, neither reference method yields good reconstruction results, especially in vulnerable regions, such as areas in *model-1* and *model-2* with similar colors in the background due to inadequate illumination. By contrast, even for poor-quality images, the proposed method can still preserve both the integrity and smoothness of the modeling surfaces.

The results of *model-4* demonstrate a failure case. All methods have limitations when parts of the model are not fully exposed, and the proposed method also failed to reconstruct an enclosed surface due to the lack of points on relevant planes. The reason is that if part of the point cloud is not dense enough relative to the other parts, after plane fitting and inlier denoising, the plane composed of these points will be separated from the *ROI*, which will be discarded after clustering, leading to incorrect reconstruction results.

**Figure 8:**
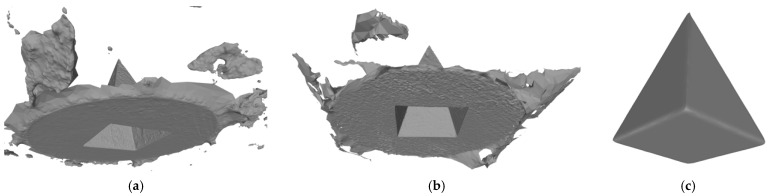
Overview of mesh bottom: (**a**) Metashape; (**b**) Meshroom; (**c**) proposed.

Another benefit of the plane fitting-based method is that the bottom surface can be reconstructed in the absence of the bottom photos as well, whereas other methods ask for manual intervention for the same purpose, as shown in Figure 8. The resulting meshes with full shapes of models can serve directly as the main graphics primitive of industrial applications such as virtual reality.

To further evaluate the validation of the proposed method in reconstructing smooth surfaces, a quantitative comparison of similarity based on the C2M (cloud-to-mesh distances) algorithm was performed. The comparative models include raw meshes yielded by the proposed feature extraction algorithm, but without plane fitting, denoised raw meshes after post-processing based on popular filtering techniques [33], and the outputs come from the proposed method. The C2M algorithm was found to be applicable on flat surfaces [45], and the comparability is presented by measuring the minimal per-vertex distance between the evaluating meshes and the ground truth models. The CloudCompare software [46,47] with a user-friendly interface was used to perform the operation. The ground truth models were reconstructed by RealityKit Object Capture [48], using images captured from the same models but with textured surfaces. In addition, to maintain the *ROI* consistency for comparability, the background noise was artificially eliminated from the relative model, and used the generated meshes without bottom populating as target meshes; thus, the models used for evaluation after the alignment of bounding-box centers and scales are given in the first column of Table 4.

The Gaussian distribution and parameters of the C2M results are also plotted in Table 4. Specifically, the smaller the mean value, the more similar the distance that the target mesh vertices are to the ground truth, while a smaller deviation indicates that the vertices are more evenly distributed on the surface; that is, the smoother the surface. The result reveals that both the post-processing of mesh filtering and the proposed one can effectively smooth the model surface. However, the proposed method has a more significant superiority, with the average of the per-vertex distances from the ground truth twice as small as that of mesh filtering, and the data distribution concentrated in a tighter interval after plane fitting and inlier denoising. The differences between the maximum and minimum distances of the proposed method are more robust among different datasets, but the post-processing results based on mesh filtering vary widely from one dataset to another.

The post-processing results for the evaluation were obtained by five iterations of mesh filtering. Although in theory the more iterations, the smoother the surface, the algorithm parameters are difficult to control [33], and as given by Table 4, even the same parameters may produce very different outcomes for different datasets. Table 5 gives a visual comparison; the first columns are post-processing results based on five and 30 iterations of mesh filtering, respectively, and the number below is the time spent for the corresponding operation in seconds. The results show that further mesh filtering is indeed more able to fill the potholes on the surface, but not only at the cost of multiplying time, but also potentially causing some areas to be over-smooth and others less smooth. In contrast, the proposed method can smooth the surface rapidly and evenly, as well as better retain the edge details.

## 5. Conclusions

Image-based 3D reconstruction of homogeneous surfaces is challenging due to the lack of distinguishable textures. To avoid the possible loss of features in vulnerable regions, a novel combination of SIFT and AKAZE was introduced to enhance robustness and accuracy. The experimental results show that the proposed method retains sufficient integrity on surfaces and edges, and is robust to illumination and affine rotation to some extent, which can help to loosen the ultimate pursuit of picture quality of traditional MVS-based 3D reconstruction. Considering that the outputs of image-based 3D reconstruction are often not directly suitable for industrial applications, with the purpose of improving the automation level, a RANSAC-based plane fitting approach was proposed to attach to the general pipeline, to classify the dense point cloud by planes and remove outliers. After projecting the inliers onto the relevant planes, all 3D points were guaranteed on an absolute plane, to facilitate the reconstruction of the *ROI* in accordance with the distribution of the points. Thanks to projection-based inlier denoising, the distance between points in each plane along the normal direction will become zero, resulting in a smooth surface. The comparison with post-processing based on mesh filtering indicates that the plane fitting-based approach outperforms the latter in both time and quality.

Although the models used in the experimental evaluation are all simulations of simple convex objects, the proposed method is applicable to any object consisting of a variety of planes. The reason is that as long as the surface points can be fitted by planes, the planar surface can be reconstructed smoothly. As shown in Table 5, both the external and internal representations of the models are well smoothed, which may be another superiority of the proposed method over the comparative method.

The main limitation of the proposed approach is that it is still heavily dependent on the capture environment, requiring a plain background and asking the object to occupy a sufficient size in the images to reduce the influence of factors such as background on the reconstruction. Another drawback is that it is time-consuming. Taking the pyramid model with 41 images as an example, the Metashape and Meshroom take about 320.23 s and 758.20 s, respectively, to achieve a complete reconstruction. However, the proposed method consumes about 1107.941 s, as it takes more time to extract and match features based on both SIFT and AKAZE. Note that even Metashape has an absolute advantage in terms of time, but running the process requires at least two manual interventions. Future work involves extending the plane fitting with other primitive fittings to enable the reconstruction of various types of homogeneous models, thus further expanding the usability of the proposed method in 3D building modeling, 3D urban object identification, and interior reconstruction. Moreover, more approaches for fostering image collection can be explored.

## Figures and Tables

**Table 1:** Specification of experimental system.

Item	Parameters
CPU	Win 10
GPU	Intel(R) Core(TM) i7—10870H@2.20 GHz
RAM	32.0 GB
HDD	SSD 953 GB

**Table 2:** Reconstruction results of models under simple background.

One of Input Images		Metashape	Meshroom	Proposed
*model-1* (n=41)	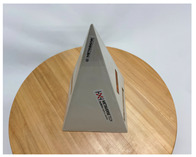	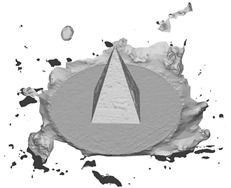	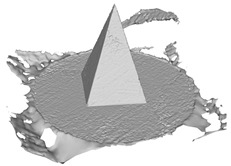	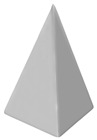
*model-2* (n=47)	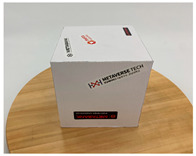	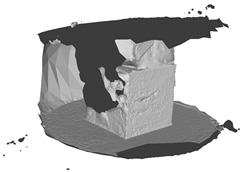	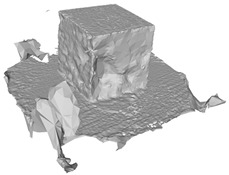	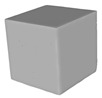
*model-3* (n=70)	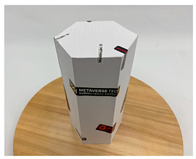	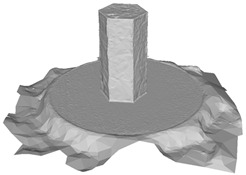	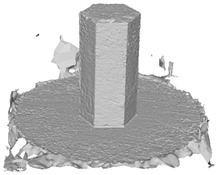	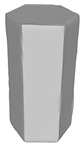
*model-4* (n=35)	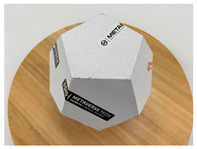	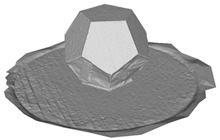	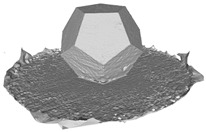	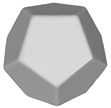

n: number of model images.

**Table 3:** Reconstruction results of models in interference-containing background.

One of Input Images		Metashape	Meshroom	Proposed
*model-1* (n=44)	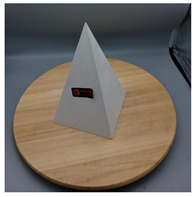	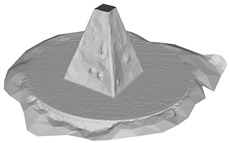	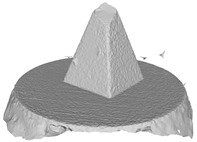	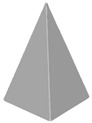
*model-2* (n=35)	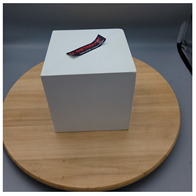	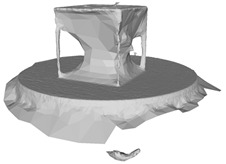	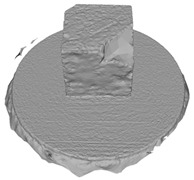	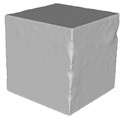
*model-3* (n=51)	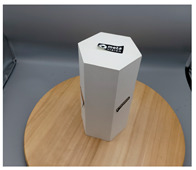	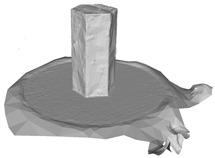	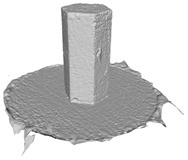	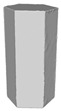
*model-4* (n=52)	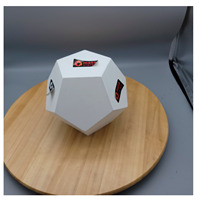	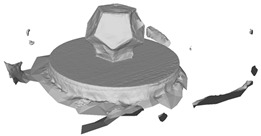	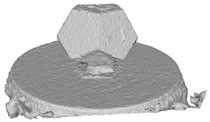	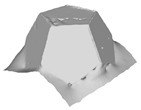

n: number of model images.

**Table 4:** C2M comparison results.

Consistency Alignment		Raw Mesh	Mesh after Filtering	Proposed
*Entry-* *1*	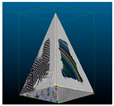	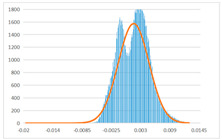	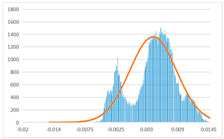	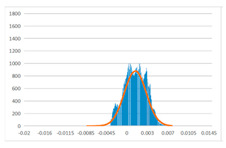
	mean	0.001764	0.001754	0.000747
	std.dev.	0.003041	0.003521	0.002044
	max−min	0.01961	0.01387	0.00827
*Entry-* *2*	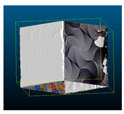	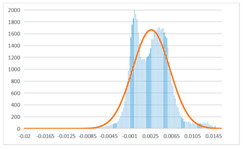	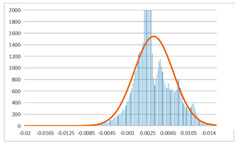	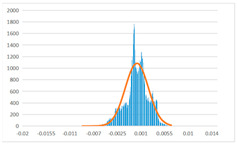
	mean	0.003746	0.002788	0.000541
	std.dev.	0.003689	0.003422	0.002105
	max−min	0.04110	0.03728	0.00936
*Entry-* *3*	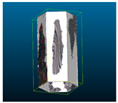	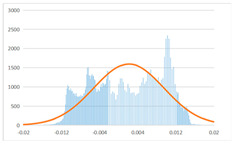	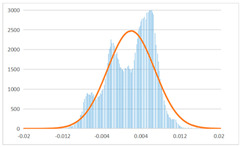	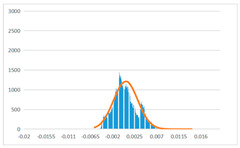
	mean	0.002133	0.001855	0.000711
	std.dev.	0.007499	0.004840	0.002470
	max−min	0.04054	0.03714	0.01412
*Entry-* *4*	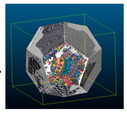	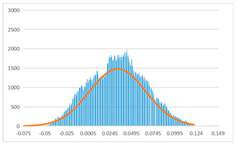	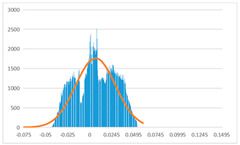	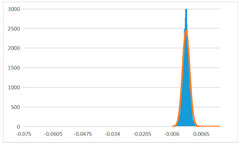
	mean	0.033621	0.006703	−0.000298
	std.dev.	0.032421	0.022685	0.001629
	max−min	0.13223	0.12155	0.01637

**Table 5:** Comparison with post-processing.

Mesh Filtering: 5 Iterations	Mesh Filtering: 30 Iterations	Proposed
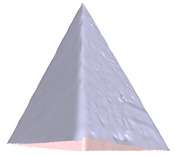	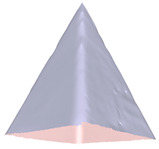	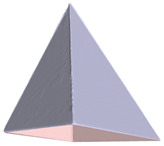
109.679	584.790	0.026
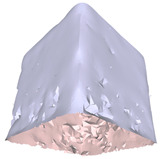	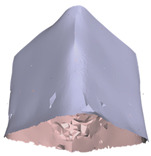	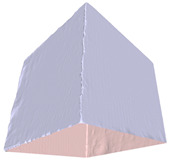
50.754	231.88	0.024
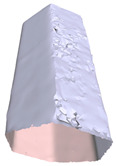	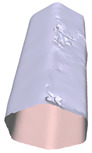	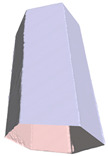
103.662	572.385	0.030
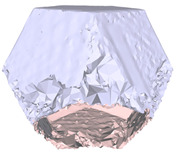	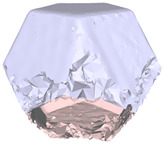	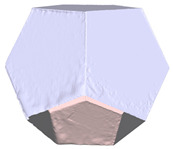
236.630	1380.929	0.034

## Data Availability

Not applicable.
